# Predictors of unfavorable responses to therapy in rifampicin-sensitive pulmonary tuberculosis using an integrated approach of radiological presentation and sputum mycobacterial burden

**DOI:** 10.1371/journal.pone.0257647

**Published:** 2021-09-20

**Authors:** Narendran Gopalan, Vignes Anand Srinivasalu, Ponnuraja Chinnayan, Banurekha Velayutham, Adhin Bhaskar, Ramesh Santhanakrishnan, Thirumaran Senguttuvan, Sridhar Rathinam, Mahilmaran Ayyamperumal, Kumar Satagopan, Dhanalakshmi Rajendran, Tamizhselvan Manoharan, Sekar Lakshmanan, Paulkumaran Paramasivam, Dhanalakshmi Angamuthu, Mangalambal Ganesan, John Washington Easudoss Arockia, Ramesh Babu Venkatesan, Venkatesan Lakshmipathy, Shivakumar Shanmugham, Balaji Subramanyam, Shakila Shankar, Jawahar Mohideen Shaheed, Baskaran Dhanaraj, Narayanan Paranji Ramiyengar, Soumya Swaminathan, Padmapriyadarsini Chandrasekaran

**Affiliations:** 1 Department of Clinical Research, National Institute for Research in Tuberculosis (formerly Tuberculosis Research Centre), Indian Council of Medical Research, Chennai, Tamil Nadu, India; 2 Department of Thoracic Medicine, Government Hospital of Thoracic Medicine Tambaram, Chennai, Tamil Nadu, India; 3 Department of Thoracic Medicine, Institute of Thoracic Medicine, Madras Medical College, Chennai, Tamil Nadu, India; 4 Chief Scientist, World Health Organization, Geneva, Switzerland; Rutgers Biomedical and Health Sciences, UNITED STATES

## Abstract

**Introduction:**

Despite the exalted status of sputum mycobacterial load for gauging pulmonary tuberculosis treatment and progress, Chest X-rays supplement valuable information for taking instantaneous therapeutic decisions, especially during the COVID-19 pandemic. Even though literature on individual parameters is overwhelming, few studies have explored the interaction between radiographic parameters denoting severity with mycobacterial burden signifying infectivity. By using a sophisticated approach of integrating Chest X-ray parameters with sputum mycobacterial characteristics, evaluated at all the three crucial time points of TB treatment namely pre-treatment, end of intensive phase and completion of treatment, utilizing the interactive Cox Proportional Hazards model, we aimed to precisely deduce predictors of unfavorable response to TB treatment.

**Materials and method:**

We extracted de-identified data from well characterized clinical trial cohorts that recruited rifampicin-sensitive Pulmonary TB patients without any comorbidities, taking their first spell of anti-tuberculosis therapy under supervision and meticulous follow up for 24 months post treatment completion, to accurately predict TB outcomes. Radiographic data independently obtained, interpreted by two experienced pulmonologists was collated with demographic details and, sputum smear and culture grades of participants by an independent statistician and analyzed using the Cox Proportional Hazards model, to not only adjust for confounding factors including treatment effect, but also explore the interaction between radiological and bacteriological parameters for better therapeutic application.

**Results:**

Of 667 TB patients with data available, cavitation, extent of involvement, lower zone involvement, smear and culture grade at baseline were significant parameters predisposing to an unfavorable TB treatment outcome in the univariate analysis. Reduction in radiological lesions in Chest X-ray by at least 50% at 2 months and 75% at the end of treatment helped in averting unfavorable responses. Smear and Culture conversion at the end of 2 months was highly significant as a predictor (p<0.001). In the multivariate analysis, the adjusted hazards ratios (HR) for an unfavorable response to TB therapy for extent of involvement, baseline cavitation and persistence (post treatment) were 1.21 (95% CI: 1.01–1.44), 1.73 (95% CI: 1.05–2.84) and 2.68 (95% CI: 1.4–5.12) respectively. A 3+ smear had an HR of 1.94 (95% CI: 0.81–4.64). Further probing into the interaction, among patients with 3+ and 2+ smears, HRs for cavitation were 3.26 (95% CI: 1.33–8.00) and 1.92 (95% CI: 0.80–4.60) while for >2 zones, were 3.05 (95% CI: 1.12–8.23) and 1.92 (95% CI: 0.72–5.08) respectively. Patients without cavitation, zonal involvement <2, and a smear grade less than 2+ had a better prognosis and constituted minimal disease.

**Conclusion:**

Baseline Cavitation, Opacities occupying >2 zones and 3+ smear grade individually and independently forecasted a poorer TB outcome. The interaction model revealed that Zonal involvement confined to 2 zones, without a cavity and smear grade up to 2+, constituting “minimal disease”, had a better prognosis. Radiological clearance >50% along with smear conversion at the end of intensive phase of treatment, observed to be a reasonable alternative to culture conversion in predicting a successful outcome. These parameters may potentially take up key positions as stratification factors for future trials contemplating on shorter TB regimens.

## Introduction

Tuberculosis (TB), the leading cause of mortality by any single infectious agent, has been evading the human immune system for centuries [1]. Utility of Chest X-rays (CXR), a simple diagnostic tool, has resurfaced in a big way during the corona pandemic, playing a pivotal role in early diagnosis, treatment transition, providing vital clues for impending complications and residual impairment in pulmonary TB [2,3].

CXR manifestations are inherently capable of segregating minimal from advanced forms of pulmonary TB, and could be used to predict treatment outcomes in pulmonary TB. The interesting study by Imperial et al., that focused on trials aimed at shortening of TB treatment, used this strategy of ‘stratified medicine approach’, to re-establish non-inferiority among the shorter and conventional six-months regimens, when they confined their pooled analysis to minimal TB disease. Minimal disease was defined in that study as a non-cavitary presentation in CXR or a smear grade of less than 2+, and without HIV co-infection [4,5]. Baseline characteristics of CXR presentations and their evolution with treatment over time had been capitalized by physicians for deciphering prognosis [6–9]. Perusal of mycobacterial load in the form of smear and culture grades at baseline and their time of conversion to negativity, is vital for a successful cure, even though the latter is time consuming [4,9]. Radiological predictors score over their bacteriological counterparts especially for taking instantaneous decisions while transiting phases or stopping treatment.

Albeit several advantages offered by CXRs in TB outcome prediction, special situations warrant careful introspection. Adult (post-primary) or childhood (primary) TB vary in their presentation and co-morbidities like HIV modify the entire radiological spectrum of TB [10]. Retreatment cases usually reveal previous scars, or calcified lesions obscuring the underlying lung architecture [8,11]. Most studies have looked into radiological predictors and mycobacterial determinants like sputum smear or culture grading in isolation without exploring the combined impact, while determining outcome, an approach routinely followed by clinicians. Hence, exploring the interplay of these two components namely radiological determinants and mycobacterial burden, that too at all the three different crucial time points of TB treatment fortified our analyses, making it not only more comprehensive statistically but a more practical approach towards assessing disease severity that dictates treatment outcome. Our ultimate destination was to arrive at clinically relevant predictors, to be applied to clinical trials for stratification, especially when shorter regimens of ATT are contemplated [12]. Our retrospective analysis of a pure clinical trial cohort of culture-confirmed rifampicin-sensitive TB patients, taking their first spell of ATT without comorbidities and meticulously followed for 24 months post treatment completion, increased the validity of conclusions drawn.

## Methodology

This retrospective de-identified individual patient data analysis was conducted on clinical trials that focused on shorter TB regimens at the Tuberculosis Research Centre, (now ICMR-National Institute for Research in Tuberculosis) Chennai, India. All trial participants had culture-confirmed, rifampicin-sensitive, newly diagnosed pulmonary TB, and without any comorbid conditions like diabetes mellitus or HIV. Treatment was fully supervised, and patients were meticulously followed up to 24 months post treatment. A brief summary of the findings of the two studies is provided in section 1.2 of the supplementary material and the complete description of trial participants is available in the main manuscript of these published trials [13,14]. CXRs, standard postero-anterior view, were taken in Akimbo’s position in the erect posture, available from baseline till end of treatment, from the per-protocol population of the two clinical trials were excavated from the radiology department, independently interpreted and subsequently collated with de-identified patient data derived from these trials, by independent statisticians (Details of regimens elaborated in e-Supplement, Section 1.1 and in Table 1). All participants in the parent trials had given their individual written informed consent and the approval of the Institutional Human Ethics Committee of Tuberculosis Research Centre (Now ICMR-NIRT) was duly obtained. Additional details provided in online supplement.

**Table 1:** Baseline characteristics of trial participants (n = 667), segregated by regimens.

Demographic Parameters	Treatment regimens					p value
	4I n = 164	2D/2I n = 99	2D/2IE n = 103	4D n = 95	6I n = 206	
**Duration of regimen in months**	4	4	4	4	6	
**Age in years** 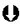	30 (23–40)	34 (24–45)	33 (24–45)	35(24–45)	31 (23–42)	0.201
**Male n (%)**	118 (71.95)	72 (72.73)	72 (69.90)	72 (75.79)	153 (74.27)	0.888
**Weight in kilograms** 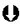	42.85 (39.78–47.58)	42.30 (38.50–48.30)	43.00 (38.00–48.90)	43.40 (38.20–48.60)	42.50 (38.88–47.60)	0.990
**Sputum Smear Grade for AFB n (%)**						
**1+**	33 (20.12)	16 (16.16)	20 (19.42)	19 (20.00)	53 (25.73)	0.652
**2+**	90 (54.88)	54 (54.55)	57 (55.34)	50 (52.63)	110 (53.40)	
**3+**	41 (25.00)	29 (29.29)	26 (25.24)	26 (27.37)	43 (20.87)	
**Sputum Culture Grade of *Mycobacterium Tuberculosis* n (%)**						
**1+**	3 (1.83)	5 (5.05)	9 (8.74)	3 (3.16)	8 (3.88)	0.267
**2+**	32 (19.51)	25 (25.25)	20 (19.42)	21 (22.11)	48 (23.30)	
**3+**	129 (78.66)	69 (69.70)	74 (71.84)	71 (74.74)	150 (72.82)	
**X-ray zones involved n (%)**						
**1**	30 (18.29)	14 (14.14)	17 (16.50)	12 (12.63)	42 (20.39)	0.463
**2**	46 (28.05)	30 (30.30)	27 (26.21)	33 (34.74)	53 (25.73)	
**3**	42 (25.61)	25 (25.25)	29 (28.16)	18 (18.95)	61 (29.61)	
**4**	30 (18.29)	16 (16.16)	18(17.48)	13 (13.68)	30 (14.56)	
**5**	10 (6.10)	8 (8.08)	8 (7.77)	8 (8.42)	12 (5.83)	
**6**	6 (3.66)	6 (6.06)	4 (3.88)	11 (11.58)	8 (3.88)	
**Number of zones** 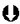	3 (2–4)	3 (2–4)	3 (2–4)	3 (2–4)	3 (2–3)	0.585
**Cavity present n (%)**	41 (25.00)	39 (39.39)	35 (33.98)	25 (26.32)	59 (28.64)	0.084
**Bilateral Lesion n (%)**	91 (55.48)	51 (51.52)	54 (52.42)	51 (53.68)	109 (52.91)	0.974
**Involvement of Lower zones n (%)**	48 (29.27)	43 (43.43)	36 (34.95)	35 (36.84)	68 (33.01)	0.205
**Co-existing non-parenchymal lesion n (%)**	17 (10.37)	13 (13.13)	15 (14.56)	13 (13.68)	24 (11.65)	0.849
**Collapse present n (%)**	6 (3.66)	3 (3.03)	5 (4.85)	3 (3.16)	3 (1.46)	0.456

n- Number, %-percentage, 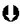
- Median (Inter Quartile Range), AFB- Acid fast bacilli. Regimens: 4I - 4 months intermittent, 2D/2I - 2 months daily followed by 2 months intermittent, 2D/2IE—2 months daily followed by 2 months intermittent with ethambutol given in continuation phase also, 4D - 4 months daily, 6I - 6 months intermittent (Standard of care). All intermittent doses were administered thrice weekly. p value—calculated probability.

### Interpretation of chest X-rays

CXRs at baseline, end of intensive phase (two months) and end of ATT, were serially and independently read by two pulmonologists possessing at least 20 years of experience in TB management with the umpire reader (more than 25 years’ experience) making the final interpretation, in case of a discrepancy. The readers had no access to clinical records or sputa results. The standard way of subdividing the lung fields into six zones, three on each side was followed (e-Supplement: S1 Fig). A detailed description of the methodology of CXR interpretation is provided in e-Supplement, Section 2.0. Conversely, the team that perused the study data, was blinded to the radiological findings. Both the data were subsequently collated and analyzed by a group of independent statisticians and finally interpreted in consensus with all physicians.

### TB outcomes

Standard clinical trial definitions were followed for TB outcomes (e-Supplement, Section 3.0). An outcome was termed favourable if all the three sputa (cultures) collected in the last month of Anti-Tubercular Treatment (ATT) were negative for *Mycobacterium tuberculosis* by Lowenstein-Jensen method and continued to be negative till 24 months post-treatment, without any re-introduction of ATT in between. Unfavorable response, in this analysis, was a composite measure that comprised of failures, recurrences and deaths.

### Statistical methods

Data were analyzed using IBM SPSS version 25.0 (IBM Corp. released 2017) and R software version 4.0.4 (R Core Team (2021). Baseline pre-treatment demographic characteristics (quantitative variables) of patients, provided as median with interquartile range, were compared for homogeneity across regimens using Kruskal-Wallis test and proportions using Chi-square test. Sputa smear and culture grades at baseline and at the end of the intensive phase were correlated with the radiological presentation corresponding to that particular time point, using Spearman rank correlation coefficient. Kaplan-Meier curves were plotted to illustrate the time to occurrence of unfavorable responses w.r.t relevant radiological parameters like cavity, radiological extent and lower zone involvement, in order to show their impact on TB outcome, with data censored at 24 months post treatment or whenever events occurred. Univariate analysis was performed, incorporating potential factors that could influence TB outcome using Chi-square test or Mann-Whitney U test. Those factors, significant at the 10% level in the univariate analysis, were fitted into the multivariable Cox proportional hazards model to calculate the adjusted hazards ratio (HR) for each factor, after adjusting for regimen and other relevant parameters at each time point namely baseline, at the end of intensive phase and end of ATT. The interaction between smear and culture grades denoting infectivity with Cavity, extent of involvement (greater than two zones) and reduction of lesions signifying severity was also studied using the same model.

## Results

Of 667 available CXRs, 449 (67%) were hard copy films while the rest were soft copies. The regimen-wise demographic profile of the participants, pre-treatment, is provided in Table 1, which showed homogeneity across the treatment arms.

**Fig 1:**
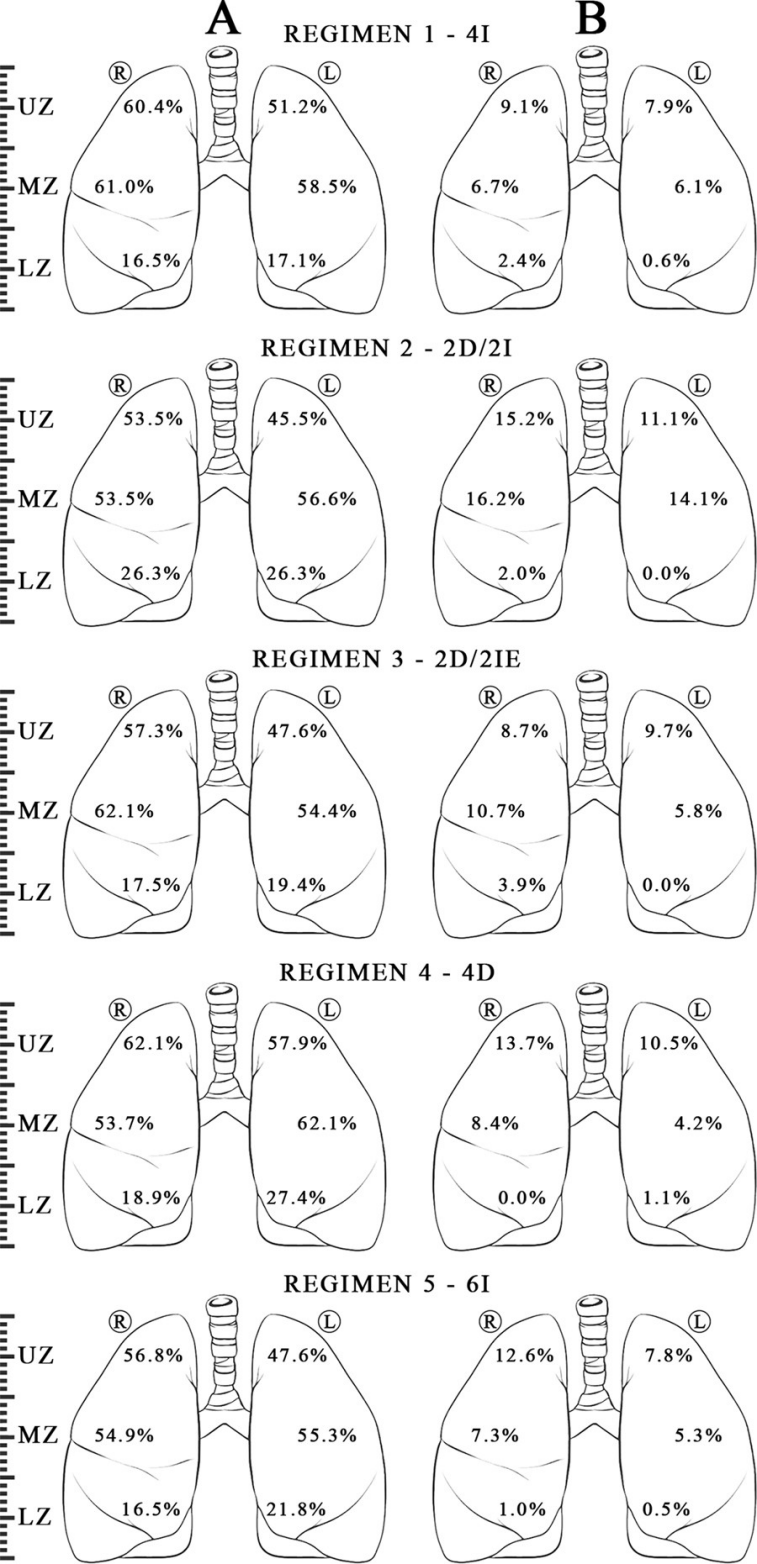
Baseline zonal distribution of lesions A -opacities and B- cavities (in percentages) shown regimen-wise. Ⓡ—Right Lung, Ⓛ–Left Lung. UZ—Upper Zone, MZ—Middle Zone, LZ—Lower Zone. Regimens - 4I - 4 months intermittent, 2D/2I - 2 months daily followed by 2 months intermittent, 2D/2IE—2 months daily followed by 2 months intermittent with ethambutol given in continuation phase also, 4D - 4 months daily, 6I - 6 months intermittent (Standard of care). All intermittent regimens were administered thrice weekly. Details of regimen provided in e-Supplement Section 1.1.

Among smear-positive, culture-confirmed pulmonary TB taking their first spell of ATT, the predominating TB lesion was opacity [96.85% (646/667)], followed by cavity [29.83% (199/667)]. Other minor manifestations included mediastinal adenopathy (5.99%, 40/667), collapse (2.99%, 20/667) and miliary nodules (1.79%, 12/667). Bilateral TB lesions accounted for 53.37% (356/667) while lower lung zone involvement was seen in 34.48% (230/667) of cases. The zonal distribution of opacities and cavities is depicted in Fig 1.

Sputum smear grade at baseline and number of zones affected, showed a weak but significant correlation (r = 0.171, p<0.001); with the baseline culture grade not exhibiting any such relationship (r = 0.071, p = 0.067). Similarly, at the end of intensive phase of treatment, the radiological clearance of lesions correlated weakly but significantly with second month smear grade (r = -0.189, p<0.001).

**Fig 2:**
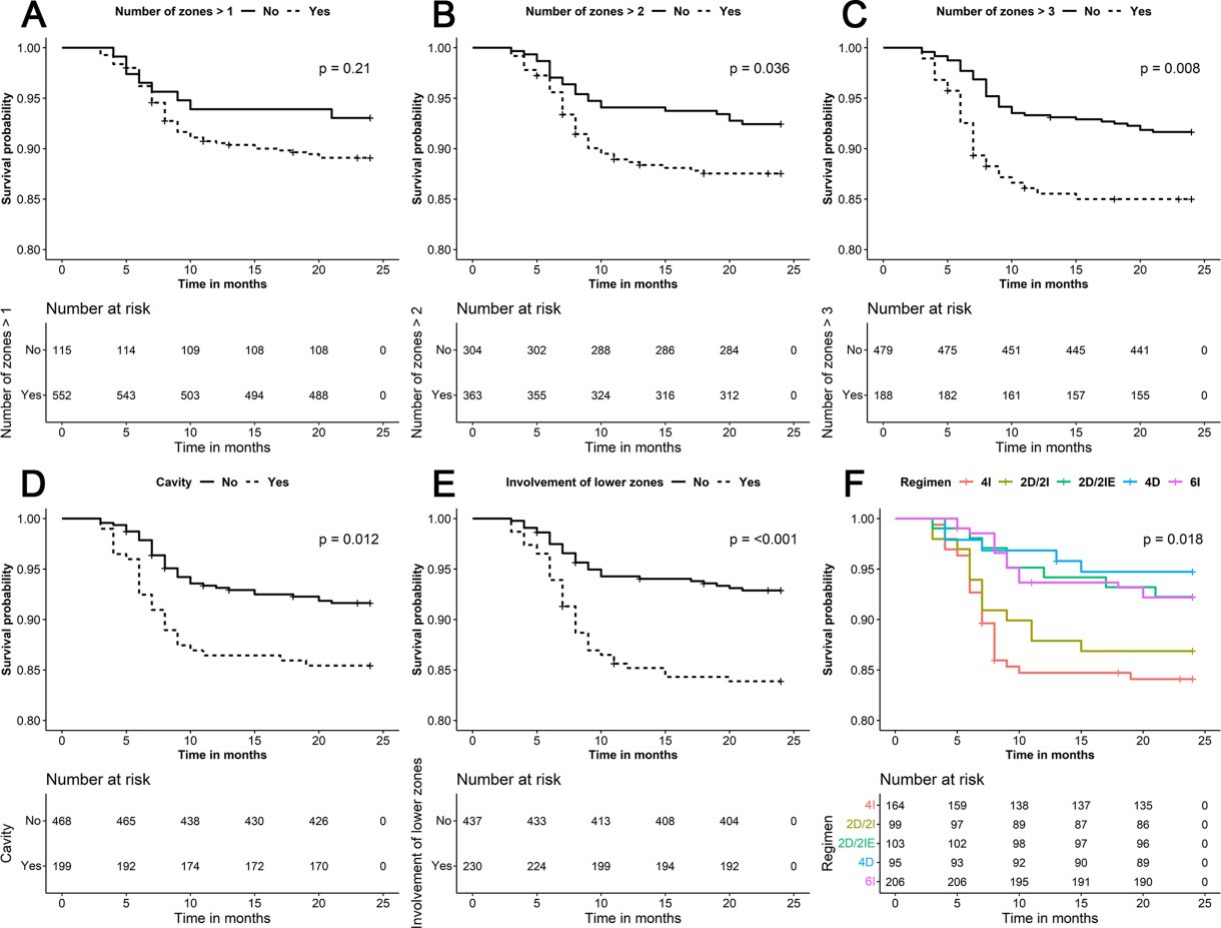
Kaplan-Meier Mean Survival Estimates for “Time to an Unfavourable Event” graphs A, B and C denote increasing order of zonal involvement, graph D—cavity present and absent, graph E—involvement of lower zones and graph F—regimen-wise distribution of unfavourable responses. >—Greater than, <—Lesser than, p–calculated probability. There was a significant difference in survival probability when the number of zones exceeded 2, there was presence of cavity or lower zone involvement and between regimens by the Log Rank (Mantel-Cox) test. The numbers given within parentheses reflects the number of individuals at risk for that factor studied, available at that particular time point.

Kaplan-Meier curves plotted to provide a bird’s eye view of the regimens, crucial factors, and their impact on TB treatment outcome, are shown in Fig 2.

Regimens - 4I - 4 months intermittent, 2D/2I - 2 months daily followed by 2 months intermittent, 2D/2IE—2 months daily followed by 2 months intermittent with ethambutol given in continuation phase also, 4D - 4 months daily, 6I - 6 months intermittent (Standard of care). All intermittent regimens were administered thrice weekly. Regimen description is provided in the e-Supplement Section 1.1

The various factors, potentiating an unfavorable response to TB therapy, at baseline, end of intensive and continuation phase, considered in the univariate analysis are enumerated in Table 2.

**Table 2:** Univariate analysis of factors influencing TB Treatment outcome.

Baseline Demographics	Categories	Unfavourable response n (%)	p value
Gender	Female	12 (6.67)	0.067
	Male	56 (11.50)	
**Baseline radiological parameters in Chest X-ray**			
Extent of involvement (Lung zones)	≤2 zones	23 (7.57)	0.007*
	>2 zones	45 (12.40)	
Bilateral involvement of lesions	No	27 (8.68)	0.227
	Yes	41 (11.52)	
Lower zone involvement	No	31 (7.09)	<0.001*
	Yes	37 (16.09)	
Co-existing non parenchymal lesions	No	59 (10.09)	0.803
	Yes	9 (10.98)	
Presence of Cavity	No	39 (8.33)	0.015*
	Yes	29 (14.57)	
Presence of Collapse^±^	No	66 (10.20)	1.000
	Yes	2 (10.00)	
**At the end of intensive phase**			
50% reduction in X ray lesion from baseline	No	40 (13.07)	0.024*
	Yes	28 (7.76)	
Presence of cavity at the end of IP	No	49 (8.97)	0.027*
	Yes	19 (15.70)	
**At the end of treatment** ^**#**^			
75% reduction in X-ray lesion from end of IP	No	47 (10.44)	0.005*
	Yes	8 (3.92)	
Presence of cavity at the end of treatment	No	43 (7.21)	0.002*
	Yes	12 (20.69)	
**Treatment regimens** 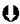			
	4I	26 (38.24)	0.025*
	2D/2I	13 (19.12)	
	2D/2IE	8 (11.76)	
	4D	5 (7.35)	
	6I	16 (23.53)	
**Bacteriological parameters at baseline**			
Sputum smear grade for AFB	1+/2+	42 (8.37)	0.006*
	3+	26 (15.75)	
Sputum culture grade for AFB	1+/2+	10 (5.75)	0.024*
	3+	58 (11.76)	
**Bacteriological parameters at end of Intensive phase**			
Sputum smear conversion at two months	No	40 (16.33)	< 0.001*
	Yes	28 (6.64)	
Sputum culture conversion at two months	No	30 (50.00)	< 0.001*
	Yes	38 (6.26)	

n—number, %—percentage, ≤—Less than or equal to, >—greater than, AFB—Acid Fast Bacilli.

*Significance was taken at the p value of 0.1 or less. ±- fishers Exact T test used. 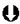
Regimens: 4I - 4 months intermittent, 2D/2I - 2 months daily followed by 2 months intermittent, 2D/2IE—2 months daily followed by 2 months intermittent with ethambutol given in continuation phase also, 4D - 4 months daily, 6I - 6 months intermittent (standard of care). All intermittent doses were administered thrice weekly.

^#^N = 654 only.

**Fig 3:**
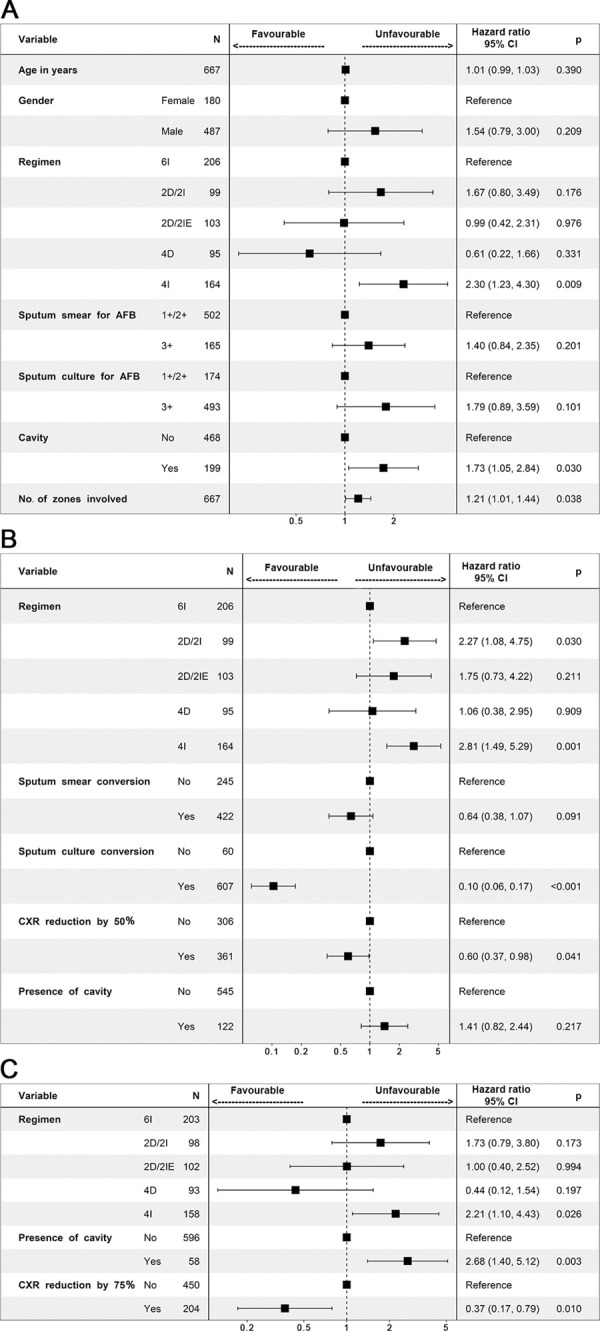
Cox Regression Model depicted in the Forest Plot showing the adjusted hazards ratio (with 95% CI and p value) of radiological and bacteriological parameters at baseline along with demographics (A), at the end of intensive phase of two months (B), at the end of treatment (C). Sample size (n) for A and B was 667, while for C, n was 654, as only recurrences were relevant to be considered at this time point for prediction. The drift towards left and right signifies tendency towards favourable and unfavourable response for each factor studied. Apart from regimen, presence of cavitation and number of zones at baseline, sputum culture conversion and CXR reduction of lesions by 50% at the end of intensive phase, persistence of cavity and CXR clearance of lesion by 75% from end of intensive phase CXR were all significantly influencing TB outcome. N—number, %—percentage, CI–Wald’s confidence interval, p-probability value (significance taken as < 0.05), AFB- acid fast Bacilli, No.—Number, CXR-Chest X-ray Regimens: 4I - 4 months intermittent, 2D/2I - 2 months daily followed by 2 months intermittent, 2D/2IE—2 months daily followed by 2 months intermittent with ethambutol given in continuation phase also, 4D - 4 months daily, 6I - 6 months intermittent (standard of care). All intermittent regimens were administered thrice weekly. Regimen description is provided in the e-Supplement Section 1.1.

The multivariable Cox proportional hazards model at each crucial timepoint of treatment showing the adjusted hazards ratios (aHR) is presented as a forest plot (Fig 3).

The highest aHR was associated with cavity followed by extent of zonal involvement. The interaction between mycobacterial infective burden in sputum and radiological severity, (Table 3), that confers better accuracy of the Cox Proportional hazards model confirmed that Cavity, >2 zones, 3+ smear, and culture conversion independently played a definitive role in predicting treatment outcome. At the end of intensive phase, reduction of lesions by 50% accompanied by smear and culture conversion showed a 74% and 95% probability of less chance of culminating in an unfavorable event respectively.

**Table 3:** Cox proportional hazards interactive model probing into the combined influence of radiological and bacteriological parameters in predicting an unfavourable response: At baseline and end of intensive phase*.

Baseline Radiological Parameter	Baseline Bacteriological Parameter	Hazards Ratio and 95% Confidence Interval	p value
**Cavity**	**Smear**		
Yes	3+	3.26 (1.33, 8.00)	0.010
	2+	1.92 (0.80, 4.60)	0.144
	1+	1.30 (0.28, 6.18)	0.738
No	3+	1.94 (0.81, 4.64)	0.135
	2+	1.05 (0.46, 2.42)	0.906
	1+	Reference	..
**Number of Zones**	**Smear**		
>2 zones	3+	3.05 (1.13, 8.24)	0.028
	2+	1.92 (0.73, 5.08)	0.188
	1+	1.55 (0.45, 5.37)	0.492
≤2 zones	3+	2.26 (0.74, 6.95)	0.153
	2+	1.04 (0.35, 3.03)	0.949
	1+	Reference	..
**Radiological parameter at the end of IP**	**Bacteriological parameter at the end of IP**	**Hazards Ratio and 95% Confidence Interval**	**p value**
**Radiological Clearance**	**Smear Conversion**		
Reduction ≥50%	Yes	0.26 (0.14, 0.49)	<0.001
	No	0.44 (0.22, 0.86)	0.017
Reduction <50%	Yes	0.30 (0.15, 0.59)	<0.001
	No	Reference	..
**Radiological Clearance**	**Culture Conversion**		
Reduction ≥50%	Yes	0.05 (0.02, 0.10)	<0.001
	No	0.63 (0.30, 1.30)	0.215
Reduction <50%	Yes	0.10 (0.05, 0.19)	<0.001
	No	Reference	..

>—greater than, ≤—less than or equal to, ≥—greater than or equal to, <—lesser than, IP—Intensive Phase.

*All interactions were adjusted for treatment effect.

## Discussion

This retrospective cross-protocol analysis, is unique in that it included a pure trial cohort of treatment naïve patients whose treatment was fully supervised and followed up every month for up to 24 months post treatment completion making the data robust. Analysis of data was performed at all the three crucial time points of TB treatment to unravel the predictors and the sophisticated interactive Cox proportional Hazards model was used to integrate sputum characteristics and radiographic presentation; all of them making interpretation easy and precise.

The tenacity of cavitation in influencing TB outcome continues to reign supreme, carrying the highest hazards ratio for any single radiological parameter in CXR, contributing towards an unfavorable responses, followed by lesions occupying greater than two zones, after adjusting for varying composition of regimens and grades of mycobacterial load, We further utilized an integrated approach of combining individual sputum smear and culture grades indicating infectivity and CXR findings denoting severity, for laying down convincing parameters for segregation into minimal and advanced TB disease and forecasting treatment outcomes. Smear grades of 1+ and 2+, in the absence of cavitation or lesions not extending beyond two zones, constituted “minimal” disease with a better prognosis, while a 3+ sputum smear or baseline cavitation or involvement greater than 2 zones of CXR independently placed the participant in the “advanced TB” category, paralleling the findings of Imperial et.al and other studies as well [4,15–17].

Cavitation, the hall mark of pulmonary TB, creates an ideal nidus for mycobacteria to thrive, with a higher bacillary load [18]. Poor vascularity of the cavity, necrotic debris with a higher extracellular bacillary burden, poor regeneration of tissues, with drugs sinking into the caseum have been the contributory factors for a suboptimal response to TB therapy [19]. The Huang et al. study reported a two-fold risk of retreatment when cavitation was present at baseline [20].

It is understandable that various guidelines initially recommended extension of ATT, when cavity persisted in CXR along with smear positivity at the end of intensive phase, based on the same principle [21,22]. The OFLOTUB study ascribed their inability to achieve non-inferiority with the shorter regimen to the higher proportion of cavitary TB disease in the cohort studied [15,16]. With a visible cavity in CXR per se contributing to TB severity, and cavity size better estimated in a computed tomography scan, we did not venture further into exploring the effect of cavity size on TB outcome [6,23]. The study by Hamilton et al. proved that the persistence of cavity beyond six months of treatment predicted TB relapse [24]. We found concurrent results as well, as our trials dealt with even more shorter regimens.

Zonal extent of lesion in the CXR plays an equally vital role in deciding TB outcome. The study by Palaci et al. showed that bacillary load in sputum increased proportionately with the area of lung involved, irrespective of the presence of cavity [18]. We found a weak correlation between extent of involvement and sputum smear grade but not with culture grade similar to other studies [6]. Combining both these crucial radiological parameters, we could reasonably hypothesize that there was a definite event-free survival advantage when TB lesions were confined to <2 zones, without cavitation in the baseline CXR apart from reduction of lesions as described in Fig 3.

Bilateral lung involvement has been postulated as a prominent risk factor for treatment failure with a HR of 1.8 (95% CI: 1.0–3.1) in the Benator et al. study [25]. Inexplicably, we also found that involvement of lower zones in a CXR independently culminated in a poorer response to treatment (Table 2), although the exact reason was unclear.

Culture conversion in our cohort proved decisive in predicting subsequent TB outcome, with a 90% probability of event free cure rate at 24 months (Fig 3). Failure of sputum culture conversion with cavity persisting at the end of 2 months led to an increased incidence of failures and recurrences [25]. The Romanoswki et al. study, using individual patient data analysis, found that among HIV seronegative population enrolled in global trials, aimed at shortening TB treatment, the adjusted odds ratio of an unfavourable TB response did not differ between smear and culture positivity at 2 months [9].

Culture grade, to direct future treatment, is limited by its long duration for obtaining results, and technical issues involved in establishing such a laboratory facility. The combined analysis of Imperial et al. showed a HR of 2.2 (95% CI: 1.7–2.9) when the second month culture remained positive [4]. In our analysis, we found a 74% lesser chance of failure when there was a combination of sputum smear conversion with radiological clearance at 2 months of treatment, a reasonable surrogate for sputum culture conversion, though less accurate. Comparing CXR’s between end of intensive phase and treatment completion, a clearance of 75% was mandated for a successful outcome to follow. Disappearance of cavity, if already present at baseline, was also important, as the latter strongly predisposed to a TB recurrence [24]. That the persistence of cavity at the end of treatment rather than at the end of second month, predisposed to failure, could be explained by the fact that two months may not be sufficient for all cavities to totally disappear. Secondly, opacities coalescing and breaking down into cavities post intensive phase, was a definite perilous sign of deterioration [3].

Interestingly, the influence of any of the parameters had a lesser impact when regimens were fortified, a feature that emerged in the recently published TBTC 31 study which showed that the combined arm of moxifloxacin with rifapentine proved non-inferior to conventional regimen among all subgroups analysed, irrespective of the baseline radiological abnormalities [26].

The main strength of our analyses lies with the unique selection of the trial cohorts studied, inherently excluding factors that could mask or modify the radiological pattern of TB in CXR [10,27]. Serial reading and independent interpretation of radiological parameters, with integration of mycobacterial burden increased the precision of our findings. The background visual interface of already pre-existing lesions in the CXR among retreatment patients, was prevented as we included only newly diagnosed cases [11]. Apparent radiological worsening or Immune Reconstitution Inflammatory Syndrome (IRIS) was considerably reduced as we dealt with an immunocompetent TB population [28]. Bias was reduced considerably by independent excavation of data by two separate clinical groups, with data amalgamated and analyzed by independent statisticians.

The main limitation of the study was the inherent heterogeneity between regimens, but this has been adequately adjusted in the model and that we focused more on baseline parameters that negated the differential influence on TB outcome. Interference among radiological parameters is a possibility. That is precisely the reason why we avoided focusing much on lesions extending to lower zone, as looking at both zonal involvement and lower lung involvement simultaneously could traumatize the assumptions of the multivariable Cox proportional hazards model. The parent clinical trials excluded diabetes mellitus and HIV from enrolment, precluding exploration in this regard. These results, hence, requires to be carefully interpreted in the light of these comorbidities.

Use of intermittent control arm of six months (prevailing standard of care) compared to daily dosing, could theoretically lead to a lesser radiological clearance. Nevertheless, findings from a parallel trial conducted during the same period, comparing daily vs intermittent ATT for six months, proved that except for culture conversion, radiological clearance and smear conversion were similar at two months, irrespective of dosing schedule [29]. Through our analysis, we could only arrive at a clear demarcation between minimal and advanced disease for stratification and therapeutic purposes, assuming that the latter would encompass both moderately advanced and far advanced cases [30].

With the global community marching towards shorter TB regimens for better patient adherence and acceptance, our study assumes significance, as our findings throw light on both radiological and bacteriological parameters that could clearly predict an unfavorable response. By this approach, we have been successful in delivering the parameters both independently as well as in an integrated manner that could progress to using the “stratified medicine concept”, maximizing efficacy in advanced cases while simultaneously minimizing toxicity in minimal disease, so as to provide a successful and uneventful recovery for the TB patients overall.

## Supporting information

S1 Fig Graphical representation of lung zones. A—Right upper lung zone, B—Left upper lung zone, C—Right middle lung zone, D—Left middle lung zone, E—Right lower lung zone, F–Left lower lung zone. Line 1—a horizontal line drawn from the anterior lower end of the second ribs. Line 2—a horizontal line drawn from the anterior lower end of the fourth ribs.
(TIF)

S1 File E-Supplement. (PDF)

S2 File Anonymised raw data used in the study. (XLSX)
