# Supplementary figures and images for: Predictors of unfavorable responses to therapy in rifampicin-sensitive pulmonary tuberculosis using an integrated approach of radiological presentation and sputum mycobacterial burden
Source: PLoS One. 2021 Sep 20;16(9):e0257647. doi: 10.1371/journal.pone.0257647 (PMC8452066; doi:10.1371/journal.pone.0257647)

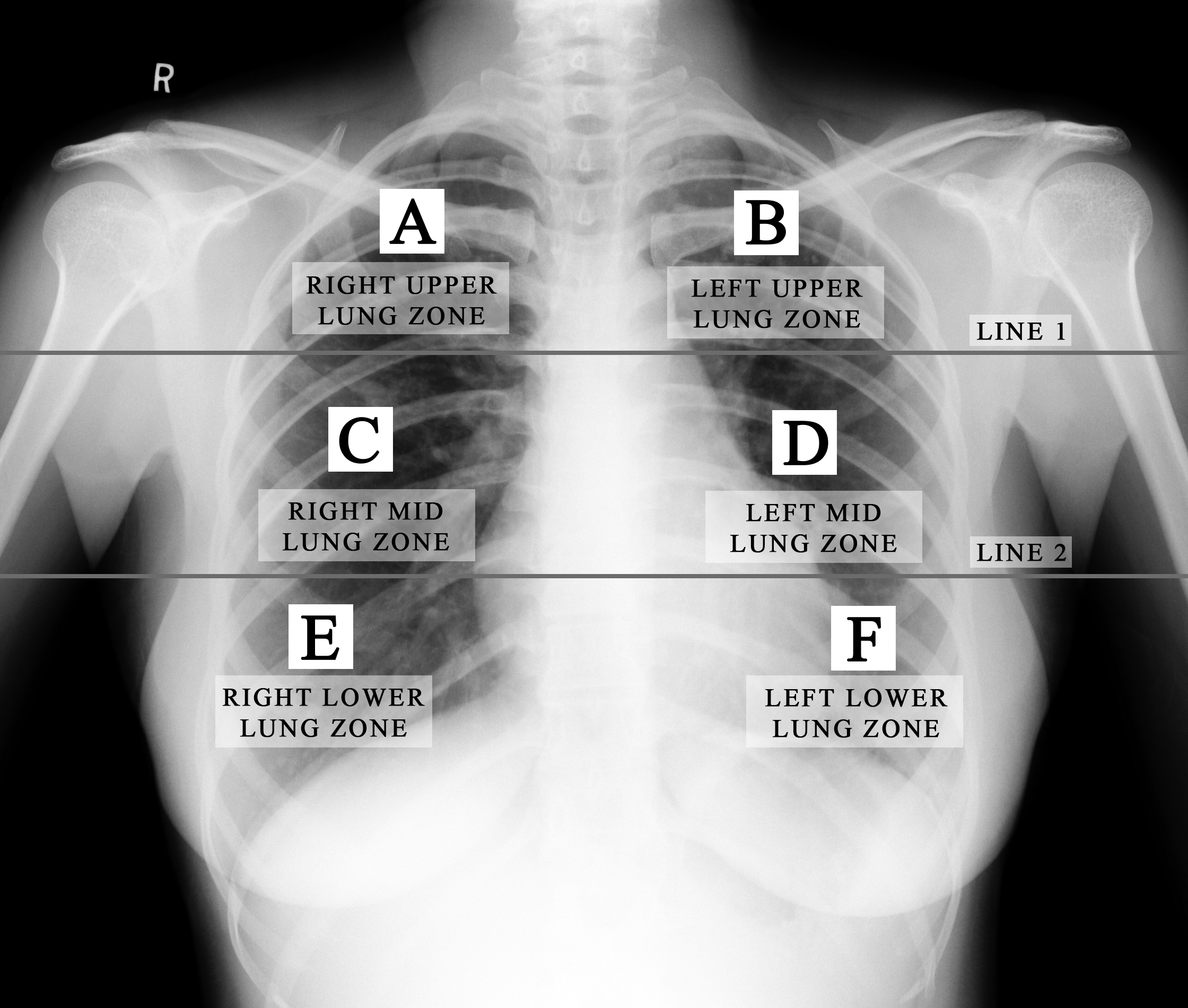

Supplement: S1 Fig — A—Right upper lung zone, B—Left upper lung zone, C—Right middle lung zone, D—Left middle lung zone, E—Right lower lung zone, F–Left lower lung zone. Line 1—a horizontal line drawn from the anterior lower end of the second ribs. Line 2—a horizontal line drawn from the anterior lower end of the fourth ribs. (TIF) [file pone.0257647.s001.tif]
