# Supplementary material for: Predictors of unfavorable responses to therapy in rifampicin-sensitive pulmonary tuberculosis using an integrated approach of radiological presentation and sputum mycobacterial burden
Source: PLoS One. 2021 Sep 20;16(9):e0257647. doi: 10.1371/journal.pone.0257647 (PMC8452066; doi:10.1371/journal.pone.0257647)
Supplement: S1 File — (PDF) [file pone.0257647.s002.pdf]

E-Supplement

Section 1.0: Study Regimens and their description.

1.1 S1 Table 1. Study regimens.

S.No.	Regimen Symbol	Regimen (IP / CP)*	Description
1	4I	2 $GHRZ_3$ / 2 GHR_3 or 2 $MHRZ_3$ / 2 MHR_3	A 4 months' fully thrice weekly regimen, containing later generation fluoroquinolones - Gatifloxacin or Moxifloxacin along with Rifampicin, Isoniazid given throughout, with the addition of Pyrazinamide in the intensive phase alone
2	2D / 2I	2 $RHZEM_7$ / 2 RHM_3	A 4 months' part daily regimen, containing daily dosing of Moxifloxacin, Rifampicin, Isoniazid, Pyrazinamide and Ethambutol in the intensive phase of first two months and a thrice weekly dosing of Moxifloxacin, Rifampicin, Isoniazid in the continuation phase for the next 2 months
3	2D / 2IE	2 $RHZEM_7$ / 2 $RHEM_3$	A 4 months' part daily regimen of Moxifloxacin, Rifampicin, Isoniazid, Pyrazinamide and Ethambutol given daily in the intensive phase and a thrice weekly dosing of Moxifloxacin, Rifampicin, Isoniazid and Ethambutol in the continuation phase.
4	4D	2 $RHZEM_7$ / 2 RHM_7	A 4 months' fully daily regimen, containing daily dosing of Moxifloxacin, Rifampicin, Isoniazid throughout, with the addition of Pyrazinamide and Ethambutol in the intensive phase of initial two months
5	6I	2 $HRZE_3$ / 4 RH_3	A 6 months thrice weekly regimen (control / Standard of care), used in the programme, containing Rifampicin, Isoniazid fortified with Pyrazinamide and Ethambutol in the initial intensive phase of two months

S. No. - Serial number, IP - intensive phase, CP - continuation phase, G - Gatifloxacin, M - Moxifloxacin, H - Isoniazid, R - Rifampicin, Z - Pyrazinamide, E - Ethambutol.
*The number prefixed denotes the duration of that phase in months and the number in the subscript denotes the number of doses in a given week for that phase as per conventional representation.

1.2 A brief summary of the two clinical trials that were aimed at shortening of TB treatment to 4 months, from which the cohort has been taken for analysis is given below in order to have a bird's eye view of the characteristics of patients enrolled.

Reference 1:

Jawahar MS et.al (2013) Randomized Clinical Trial of Thrice-Weekly 4-Month Moxifloxacin or Gatifloxacin Containing Regimens in the Treatment of New Sputum Positive Pulmonary Tuberculosis Patients. PLOS ONE 8(7): e67030. https://doi.org/10.1371/journal.pone.0067030.

This was a randomised control clinical trial that attempted to shorten tuberculosis (TB) treatment to 4 months using gatifloxacin or moxifloxacin in combination with other anti-tubercular treatment (ATT) drugs. Newly diagnosed, sputum-positive HIV-negative adult pulmonary TB patients were randomly allocated to receive gatifloxacin or moxifloxacin, along with isoniazid and rifampicin for 4 months with pyrazinamide for first 2 months (G or M) or isoniazid and rifampicin for 6 months with ethambutol and pyrazinamide for first 2 months (C). All regimens were administered thrice-weekly. Clinical and bacteriological assessments were done monthly during treatment and for up to 24 months post-treatment. The mean weight of the participants was 43.6 kilogram and, the sputum culture was 3+ and the number of zones in X-ray was >2 in 79% of the study participants signifying extensive disease. Second month culture negativity was 83% in the gatifloxacin arm, 88% in the moxifloxacin arm and 78% in the control arm (standard of care). The main aim of the study was comparison of relapses which was 17 (15%) of 115 in the gatifloxacin arm, 11 (11%) of 104 in the moxifloxacin arm and 8 (6%) of 132 patients in the control arm respectively. The Data and Safety Monitoring Board

recommended termination of the trial due to high TB recurrence rates observed in the G and M regimens when given intermittently. **The study concluded** that a 4-month thrice-weekly regimens of gatifloxacin or moxifloxacin with isoniazid, rifampicin and pyrazinamide, were inferior to standard 6-month treatment, in patients with newly diagnosed sputum positive pulmonary TB [13].

Reference 2:

Velayutham B et.al 4-month moxifloxacin containing regimens in the treatment of patients with sputum-positive pulmonary tuberculosis in South India - a randomised clinical trial. Trop Med Int Health. 2020 Apr;25(4):483-495. doi: 10.1111/tmi.13371. Epub 2020 Feb 3. PMID: 31944502. https://doi.org/10.1111/tmi.13371

This study, aimed to test the efficacy of 3- and 4-month regimens containing moxifloxacin in a randomised clinical trial in pulmonary tuberculosis (PTB) patients by enrolling new, sputum-positive, adult, HIV-negative, non-diabetic PTB patients. The study drugs used were moxifloxacin (M), isoniazid (H), rifampicin (R), pyrazinamide (Z) and ethambutol (E) or to a control regimen ($2H_3R_3Z_3E_3/4R_3H_3$) [C]. The 4 test regimens were $3R_7H_7Z_7E_7M_7$, $2R_7H_7Z_7E_7M_7/2R_7H_7M_7$, $2R_7H_7Z_7E_7M_7/2R_3H_3M_3$ and $2R_7H_7Z_7E_7M_7/2R_3H_3E_3M_3$. Treatment was directly observed. Clinical and bacteriological assessments were done monthly during treatment and for 24 months post-treatment. The primary end point was TB recurrence post-treatment. The baseline characteristics of the enrolled participants showed that 78% had a 3+ culture grade and 79% having more than 2 chest X-ray zones involved. Cavitation was present in 43% of cases. 2nd month culture conversion was 94% among moxifloxacin containing regimens while it was 77% in the thrice weekly control arm. Of 1329 patients enrolled in the modified intention-to-treat (ITT) analysis, 3-month daily regimen was prematurely terminated due to high TB recurrence rates. 'Favourable' response at end of treatment was 96-100% in the moxifloxacin regimens and 93% in the control regimen. Among these, the TB recurrence occurred in 4.1% in the 4 months daily regimen containing Moxifloxacin compared to 4.5% in the control regimen. **In conclusion**, the 4-month daily moxifloxacin regimen [M4] was found to be equivalent and as safe as the 6-month thrice-weekly control regimen [14].

Section 2.0: Chest X-ray (CXR) interpretation.

2.1 Selection of X-rays and interpretation

The cohort analyzed in this study was limited to those participants whose Chest X-rays were available serially for all the three decisive time points of treatment period, namely baseline or pre-treatment, 2nd month or end of intensive phase and end of treatment. All the patients were exposed to a standard Chest X-ray PA view taken in Akimbo's position, at a distance of 6 feet between the tube and the screen. All the soft copy images were of DICOM format (Digital Imaging and communications in Medicine). For valid comparison, each patient needed to have X-rays either as soft or hard copies uniformly throughout for that particular patient at all crucial time points. X-rays of poor quality were excluded.

To minimize inaccuracies in interpretation, the visual estimation was done in such a way that, those lesions with definite evidence of zonal involvement and those with visibly apparent reduction in lesion size were considered. The readers were totally blinded to the clinical data and their observations were captured in separate files and sent to the independent statistician for amalgamation. Two Readers who were pulmonologists managing TB for at least 20 years interpreted the X-rays independently with an umpire reader who had at least 25 years' experience interpreting in cases of discrepancies.

S1 Table 2. Chest X-ray zones with corresponding representative alphabets.

Alphabet	Representation
a	Right Upper Zone
b	Left Upper Zone
c	Right Mid Zone
d	Left Mid Zone
e	Right Lower Zone
f	Left Lower Zone

- ❖ The upper zone is demarcated from the Mid zone by a horizontal line drawn from the anterior lower end of the second rib.
- ❖ The Mid zone is bordered from the lower zone by a horizontal line drawn from the anterior lower end of the fourth rib.
- ❖ The zones have no relationship with the lobes of the Lung in the chest Xray.

Simple visual estimation was used, mimicking the way patients are managed at hospitals, by characterizing the lesions as shown in the table and assigning a number to each type of lesion. The zones were denoted as alphabets.

A simple picture to denote the description of zones is given below.

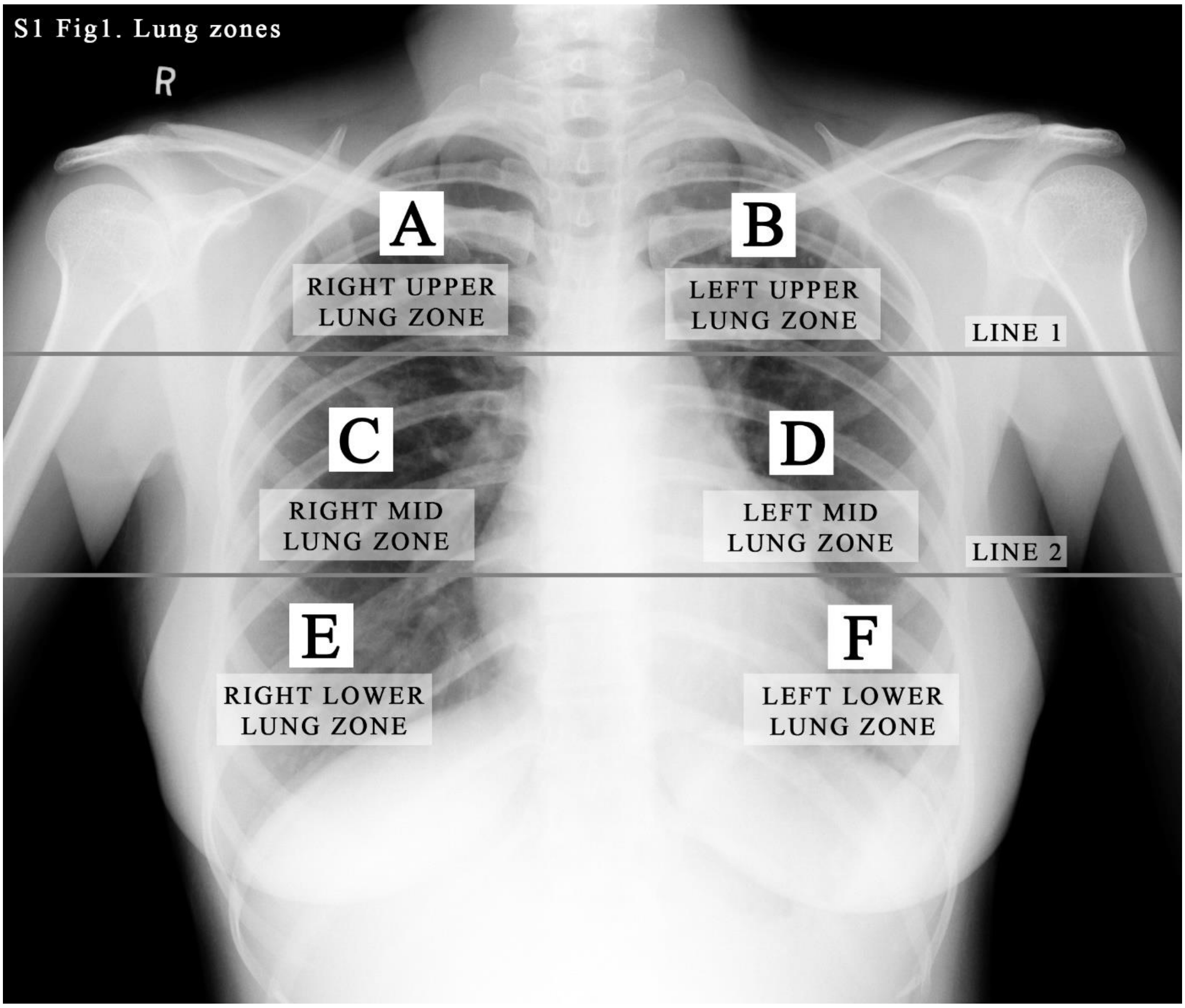

S1 Fig 1: Graphical representation of Lung Zones. A - Right upper lung zone, B - Left upper

lung zone, C - Right middle lung zone, D - Left middle lung zone, E - Right lower lung zone, F – Left lower lung zone. Line 1 - a horizontal line drawn from the anterior lower end of the second ribs. Line 2 - a horizontal line drawn from the anterior lower end of the fourth ribs.

2.2 Measurement of reduction or clearance of radiological lesion

We used a slightly modified Ralph scoring for calculating the reduction percentage of radiological lesions or radiological clearance by comparing the CXR's at two consecutive points i.e., baseline and the end of intensive phase, End of intensive phase and end of treatment. At baseline, definitive lesions in each of the zones were counted at 100% (dichotomous scoring was used, presence was 100, and absent was 0). In a similar way, the lesions in the follow-up serial X-rays at end of intensive phase (IP) and end of treatment or continuation phase (CP) were also read and recorded. An increase in size of a lesion roughly 50% from baseline size was considered deterioration (D), a visual decrease in size of a lesion by at least 50% from baseline as improvement (I), with similar picture as previous one - status quo (S). If the lesion at baseline disappeared completely in the follow-up X-ray reading at end of IP, it was termed as resolved (R). If any new lesion arises in one of the zones or any lesion extends into other neighboring zones in the follow-up X-ray at end of IP, and was previously not detected in the baseline CXR, then it was coined as newly appeared (A). This R-I-S-D-A classification and coding was followed for each lesion in each zone in the follow up X-rays and numerically represented as R (+100%), I (+50%), S (0), D (-50%) and A (-100%). Going by the pathophysiology of pulmonary TB, lesions that signified healing process of the TB disease such as fibrosis and calcifications were scored as improvement as would be deciphered by a treating clinician and given a score of 50% in the follow up X-rays. An opacity breaking down into cavitation was given a score of - 100% (minus 100%) as it signified deterioration for practical estimation, paying due heed to the Ralph scoring method. After assigning the individual scores to the topographical lesions in each zone, a simple reduction percentage was

calculated by dividing the sum of the scores of each lesion in the X-ray at end of IP or end of treatment, by the number of lesions accounted for, to arrive at the overall reduction percentage. We felt that this kind of scoring exactly adapts itself to the practical way in which clinicians decipher improvement or deterioration.

Section 3.0: Standard definitions of TB treatment outcome used in the trials

Status at the end of treatment:

a. 'Favourable' - Defined as all three sputum cultures being negative in the last month of treatment or if one culture was positive but subsequent monthly cultures were negative without additional chemotherapy. The patient continues to be sputum culture negative till 24 months post treatment.

b. 'Unfavourable' - Any of the following: 1) Unfavourable bacteriological response – Defined as more than one sputum culture being positive in the last month of treatment, one of which was at least 20 colonies or more, or one culture being positive in the last month of treatment followed by positive cultures in subsequent months, 2) Treatment was changed for persistent positive sputum cultures 3) Treatment changed for radiological or clinical deterioration or due to toxicity, 4) Patient died during treatment

All outcomes were decided by an END point review committee in an unbiased fashion when the Principal Investigator or designee presented each case.
